# One-step, kit-based radiopharmaceuticals for molecular SPECT imaging: a versatile diphosphine chelator for ^99m^Tc radiolabelling of peptides†

**DOI:** 10.1039/d1dt03177e

**Published:** 2021-10-15

**Authors:** Ingebjørg N. Hungnes, Fahad Al-Salemee, Peter J. Gawne, Thomas Eykyn, R. Andrew Atkinson, Samantha Y. A. Terry, Fiona Clarke, Philip J. Blower, Paul G. Pringle, Michelle T. Ma

**Affiliations:** King's College London, School of Biomedical Engineering and Imaging Sciences 4th Floor Lambeth Wing St Thomas’ Hospital London UK michelle.ma@kcl.ac.uk; King's College London, Randall Centre for Cell and Molecular Biophysics, and Centre for Biomolecular Spectroscopy London UK; Institut de Pharmacologie et de Biologie Structurale, IPBS, Université de Toulouse, CNRS, Université Paul Sabatier 31077 Toulouse France; King's College London, Centre for Inflammation Biology and Cancer Immunology, Faculty of Life Sciences and Medicine London UK; University of Bristol, School of Chemistry Cantock's Close Bristol UK

## Abstract

Radiotracers labelled with technetium-99m (^99m^Tc) enable accessible diagnostic imaging of disease, provided that radiotracer preparation is simple. Whilst ^99m^Tc radiopharmaceuticals for imaging perfusion are routinely prepared from kits, and regularly used in healthcare, there are no ^99m^Tc-labelled receptor-targeted radiopharmaceuticals in widespread clinical use. This is in part due to the multistep radiosyntheses required for the latter. We demonstrate that the diphosphine, 2,3-bis(diphenylphosphino)maleic anhydride (BMA), is an excellent platform for preparation of kit-based, receptor-targeted ^99m^Tc-labelled radiotracers: its conjugates are simple to prepare and can be easily labelled with ^99m^Tc using one-step, kit-based protocols. Here, reaction of BMA with the α_v_β_3_-integrin receptor targeted cyclic peptide, Arg-Gly-Asp-DPhe-Lys (RGD), provided the first diphosphine-peptide conjugate, DP-RGD. DP-RGD was incorporated into a “kit”, and addition of a saline solution containing ^99m^TcO_4_^−^ to this kit, followed by heating, furnished the radiotracer [^99m^TcO_2_(DP-RGD)_2_]^+^ in consistently high radiochemical yields (>90%). The analogous [ReO_2_(DP-RGD)_2_]^+^ compound was prepared and characterised, revealing that both [^99m^TcO_2_(DP-RGD)_2_]^+^ and [ReO_2_(DP-RGD)_2_]^+^ consist of a mixture of *cis* and *trans* geometric isomers. Finally, [^99m^TcO_2_(DP-RGD)_2_]^+^ exhibited high metabolic stability, and selectively targeted α_v_β_3_-integrin receptors, enabling *in vivo* SPECT imaging of α_v_β_3_-integrin receptor expression in mice.

## Abstract 2

We report a versatile diphosphine platform for ^99m^Tc radiolabelling of peptides. The diphosphine-peptide conjugate can be radiolabelled with ^99m^Tc in a single step, and the new radiotracer is capable of receptor-targeted imaging of disease *in vivo*.

## Graphical abstract

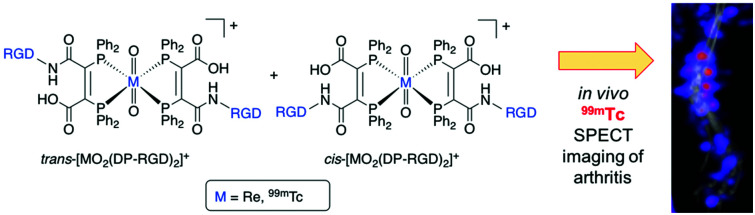

## Introduction

The γ-emitting radionuclide, technetium-99 m (^99m^Tc, *t*_1/2_ = 6 h, 90% γ, 140 keV), is used in over 30 million routine nuclear medicine SPECT/γ-scintigraphy procedures every year, for diagnostic imaging of perfusion and anatomical processes.^1,2^^99m^Tc is produced by bench-top generators, enabling this widespread access. Despite the availability of ^99m^Tc and the high prevalence of SPECT and γ-scintigraphy infrastructure, few receptor-targeted ^99m^Tc molecular imaging agents have entered late stage clinic trials, and none are used routinely. In contrast, modern PET imaging with peptide-based, receptor-targeted radiotracers has had significant clinical impact. ^68^Ga-labelled peptides that target receptors over-expressed in prostate and neuroendocrine cancers have resulted in better disease management for patients and are now used in routine clinical practice.^3,4^

^99m^Tc radiopharmaceuticals for imaging heart, kidney and brain perfusion are based on one-step, kit-based radiosyntheses, in which generator-produced ^99m^TcO_4_^−^ is simply added to commercially available “kit” vials that contain a reducing agent, chelator and other reagents.^2,5^ These simple radiosynthetic procedures allow staff in hospital radiopharmacies to routinely prepare patient doses of ^99m^Tc radiopharmaceuticals on a daily basis.

**Chart 1:**
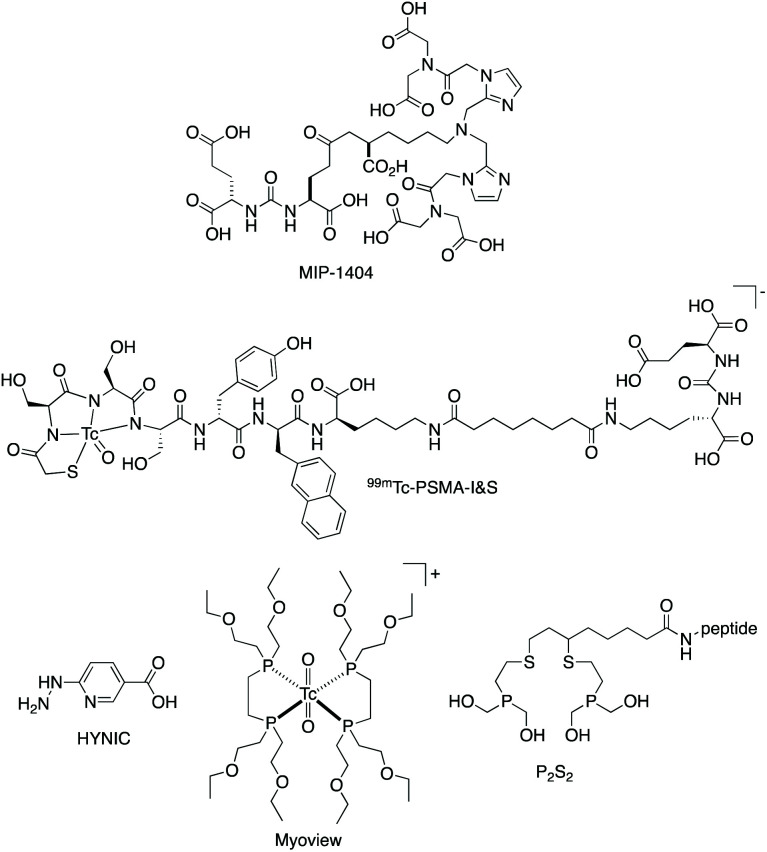
Existing chelators and complexes used for ^99m^Tc radiopharmaceuticals.

Several ^99m^Tc-labelled chelator–peptide conjugates have recently demonstrated clinical utility in SPECT imaging of receptor expression. These include ^99m^Tc-MIP-1404 and derivatives, and ^99m^Tc-PSMA-I&S, which target PSMA (prostate specific membrane antigen) receptors that are overexpressed in prostate cancer.^6,7^ In ^99m^Tc-MIP-1404, the tridentate N_3_ chelator (Chart 1) coordinates to a *fac*-[^99m^Tc(CO)_3_]^+^ moiety.^6^ In ^99m^Tc-PSMA-I&S (Chart 1), a modified tripeptide, mercaptoacetyl-d-Ser-d-Ser-d-Ser, coordinates to the [^99m^TcO]^3+^ motif, *via* a thiol and three deprotonated amide groups.^7^ These PSMA-targeted radiopharmaceuticals are prepared from kits. However, whilst ^99m^Tc-PSMA-I&S is prepared in a single step at high radiochemical yields, preparation of ^99m^Tc-MIP-1404 involves two “kits” – the first to generate the labile *fac*-[^99m^Tc(CO)_3_(H_2_O)_3_]^+^ precursor and the second to form the ^99m^Tc complex of the targeting chelator–peptide bioconjugate MIP-1404. Other molecular ^99m^Tc radiopharmaceuticals are based on 6-hydrazinopyridine-3-carboxylic acid (HYNIC, Chart 1), which coordinates to ^99m^Tc and acts as an attachment point for targeting peptides.^8^ Co-ligands such as ethylenediamine or tricine occupy remaining coordination sites on the Tc. Some of these HYNIC-based radiopharmaceuticals can be prepared from a single kit,^9,10^ but their structures remain ill-defined: it is unknown whether HYNIC coordinates to Tc *via* the hydrazino group only, or as a bidentate ligand, *via* the hydrazino and pyridyl groups.^11,12^

The radiopharmaceutical “Myoview” is used to image cardiac perfusion. In Myoview, two bidentate diphosphines coordinate to a *trans*-[TcO_2_]^+^ motif (Chart 1).^13^ Myoview is also prepared using a single step: ^99m^TcO_4_^−^ is added to a kit containing sodium gluconate, tin chloride, sodium bicarbonate and diphosphine chelator, followed by incubation at room temperature for 15 min to produce Myoview in >90% radiochemical yield and purity. It is then administered to patients without further processing.^14^ Other chelators containing phosphines, notably a P_2_S_2_ chelator (Chart 1), have also exhibited efficient radiolabelling properties when reacted with [TcO_2_]^+^ derivatives.^15–17^

**Scheme 1:**
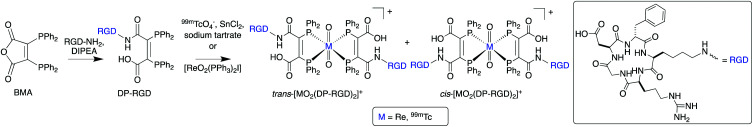
Preparation of [MO_2_(DP-RGD)_2_]^+^ (M = Re, ^99m^Tc).

We aim to identify new diphosphine chemical platforms that enable simple, one-step, kit-based ^99m^Tc-radiolabelling of receptor-targeted peptides, to provide structurally well-defined ^99m^Tc radiotracers. Prior work has shown that primary amines react with 2,3-bis(diphenylphosphino)maleic anhydride (BMA, Scheme 1) to form a ring-opened amide species.^18,19^ We have therefore selected BMA as a potentially versatile chemical platform for preparing a diphosphine-peptide conjugate. We have also selected the cyclic peptide, Arg-Gly-Asp-DPhe-Lys (RGD), which targets the α_v_β_3_-integrin receptor over-expressed in neovasculature, inflammation processes and cancer cells. RGD has been used extensively in receptor-targeted imaging,^9,20^ and it contains a single primary amine suitable for bioconjugation. We have chosen the [^99m^Tc^V^O_2_]^+^ motif for radiolabelling of diphosphine-peptide conjugates because well-defined phosphine complexes based on [^99m^Tc^V^O_2_]^+^ (*e.g.* Myoview) can be prepared from aqueous solutions of ^99m^TcO_4_^−^ in a single step. Additionally, in comparison to the commonly used [^99m^Tc(CO)_3_]^+^ motif, the [^99m^Tc^V^O_2_]^+^ group is relatively hydrophilic, and this property favours rapid radiotracer clearance from circulation *via* a renal pathway, potentially enabling high contrast SPECT imaging of target disease.

## Results and discussion

### Synthesis and radiolabelling

Reaction of RGD with BMA^21,22^ in a basic solution of DMF followed by semi-preparative reverse-phase C_18_ HPLC provided the diphosphine conjugate DP-RGD (Scheme 1) in ≥95% purity and 86% yield. DP-RGD was characterised by ^1^H, ^13^C and ^31^P NMR, analytical HPLC and HR-ESI-MS (Fig. S1–S6 and Tables S1, S2†). Whilst DP-RGD slowly oxidised in solution to phosphine oxide derivatives under normal atmospheric conditions, in the solid state, DP-RGD was stable to oxidation: it can be handled either as a dry powder, in basic organic solutions, or in aqueous solutions at near-neutral pH. However, in acidic solutions, the reverse reaction was observed, and the DP-RGD conjugate decomposed to re-form RGD and BMA.

The chemistry of Re and Tc are closely similar. As Tc has no stable isotopes, it was convenient to prepare [ReO_2_(DP-RGD)_2_]^+^ in order to obtain full characterisation. Reaction of [ReO_2_I(PPh_3_)_2_] with an excess of DP-RGD furnished geometric isomers of [ReO_2_(DP-RGD)_2_]^+^ (Scheme 1), which are labelled *cis* and *trans* to denote the relative positions of the RGD moieties, and which were formed in the ratio of 54% and 46% respectively. The isomers were separated by reverse phase C_18_ HPLC. For both species, the most intense signals in the ESI-MS at *m*/*z* = 785.92 and 1178.38, corresponded to the ions [M + 2H]^3+^ and [M + H]^2+^ where M = [ReO_2_(DP-RGD)_2_]^+^ (see Fig. S1†).

**Fig. 1:**
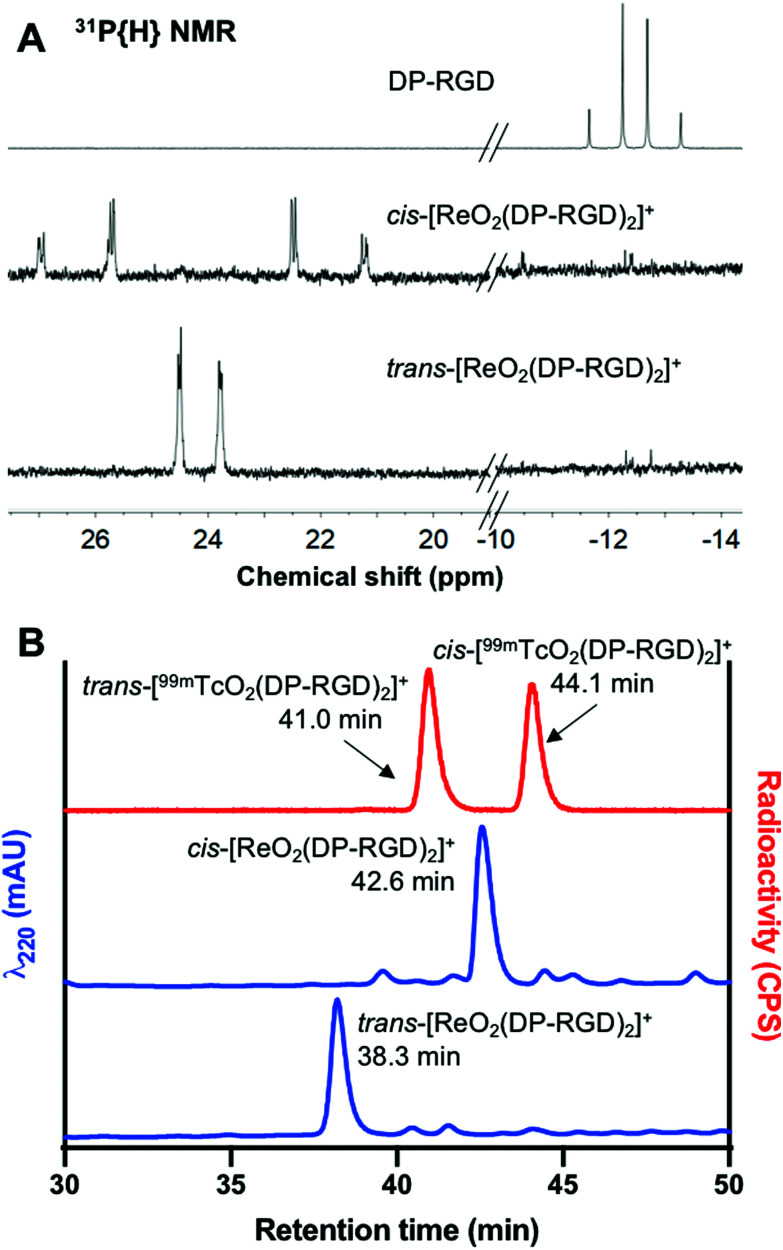
(A) ^31^P{H} NMR of DP-RGD, *cis*-[ReO_2_(DP-RGD)_2_]^+^ and *trans*-[ReO_2_(DP-RGD)_2_]^+^. The full ^31^P NMR spectra are included in Fig. S6.† (B) Radio-HPLC trace of *trans*-/*cis*-[^99m^TcO_2_(DP-RGD)_2_]^+^ (red) prepared from an aqueous solution of ^99m^TcO_4_^−^ and an optimised kit formulation (Kit 3, Table 1), and HPLC traces (λ_220_) of *trans*- and *cis*-[ReO_2_(DP-RGD)_2_]^+^ (blue). The full chromatograms are included in Fig. S7.†

In the ^31^P{H} NMR spectrum of DP-RGD (Fig. 1A), the two inequivalent P atoms produce an AB pattern with *δ*(P_A_) = −12.04 and *δ*(P_B_) = −12.89 and ^2^*J*_PP_ = 168.4 Hz. The ^31^P{H} NMR spectra of the isomers of [ReO_2_(DP-RGD)_2_]^+^ might be expected to show complex patterns associated with the AA′XX′ spin systems. However the spectra for the two isomers are shown in Fig. 1A and the upper spectrum is tentatively assigned to the *cis* isomer on the basis of the pseudo-AB pattern with a large ^2^*J*(P_A_P_B_) (356.1 Hz) expected for *trans*-inequivalent P atoms.^23,24^ In the ^1^H and ^13^C NMR spectra, the PPh_2_ signals shift upon Re^V^ binding and become more complex, but RGD peptide resonances are not changed significantly (Fig. S3–S5 and Tables S1, S2†).

To assess the feasibility of ^99m^Tc radiolabelling of DP-RGD with a “kit” formulation, lyophilised mixtures of DP-RGD, stannous chloride, sodium bicarbonate, and sodium gluconate were initially prepared (Table 1, Kit 1). The amounts of stannous chloride, sodium bicarbonate and sodium gluconate reagents used in Kit 1 replicate those in the Myoview kit. Stannous chloride reduces ^99m^TcO_4_^−^ to ^99m^Tc(V), sodium bicarbonate buffers the solution at pH 8 and sodium gluconate is a weak chelator that stabilises reduced ^99m^Tc intermediates, and also coordinates Sn^2+^ in solution, to prevent formation and precipitation of stannous hydroxide species during radiolabelling reactions. All radiolabelling reactions were undertaken in a mixture of saline and ethanol to dissolve DP-RGD; lower amounts of ethanol were required for kits containing lower amounts of DP-RGD.

**Table 1:** Materials used in kit-based reactions for preparation of [^99m^TcO_2_(DP-RGD)_2_]^+^ and Myoview

Kit	Kit components	Radiochemical yield^a^
1	DP-RGD: 1.0 mg (0.93 μmol)	≤34%
	Sodium gluconate (NaC_6_H_11_O_7_): 1.0 mg (4.58 μmol)	
	SnCl_2_·2H_2_O: 50 μg (0.22 μmol)	
	NaHCO_3_: 1.8 mg (21.43 μmol)	
	^99m^TcO_4_^−^ in 150 μL saline/150 μL EtOH	
2	DP-RGD: 500 μg (0.47 μmol)	85%
	Sodium tartrate (Na_2_C_4_H_4_O_6_): 1.05 mg (4.58 μmol)	
	SnCl_2_·2H_2_O: 50 μg (0.22 μmol)	
	NaHCO_3_: 1.8 mg (21.4 μmol)	
	^99m^TcO_4_^−^ in 150 μL saline/150 μL EtOH	
3	DP-RGD: 125 μg (0.12 μmol)	≥90%
	Sodium tartrate: 0.26 mg (1.15 μmol)	
	SnCl_2_·2H_2_O: 25 μg (0.11 μmol)	
	NaHCO_3_: 0.9 mg (10.71 μmol)	
	^99m^TcO_4_^−^ in 250 μL saline/50 μL EtOH	
4	DP-RGD: 64 μg (0.06 μmol)	65%
	Sodium tartrate: 0.26 mg (1.15 μmol)	
	SnCl_2_·2H_2_O: 25 μg (0.11 μmol)	
	NaHCO_3_: 0.9 mg (10.71 μmol)	
	^99m^TcO_4_^−^ in 260 μL saline/40 μL EtOH	
Myoview (single dose kits)	Diphosphine: 250 μg (0.65 μmol)	Routinely > 90%
	Sodium gluconate: 1.0 mg (4.6 μmol)	
	SnCl_2_·2H_2_O: 50 μg (0.22 μmol)	
	NaHCO_3_: 1.8 mg (21.4 μmol)	
	^99m^TcO_4_^−^ in saline	

a Reactions were undertaken in duplicate to ensure reproducibility of radiochemical yields, except for radiolabelling reactions with Kit 3, where the reaction was replicated four times to give an average radiochemical yield of 93.0 ± 1.0%.

Addition of generator-produced ^99m^TcO_4_^−^ in saline solution (20–55 MBq) to the contents of Kit 1, followed by heating at 60 °C for 30 min, resulted in formation of [^99m^TcO_2_(DP-RGD)_2_]^+^ in radiochemical yields of up to 34%, as determined by radio-HPLC (*vide infra*) and TLC. Replacing sodium gluconate with sodium tartrate in the “kit” mixture whilst lowering the amount of DP-RGD conjugate from 1 mg to 0.5 mg, increased radiochemical yields to 85% (Kit 2). In Kit 3, radiochemical yields of 93.0 ± 1.0% (*n* = 4) were achieved, with 45–65 MBq of ^99m^TcO_4_^−^ and only 125 μg of DP-RGD, providing specific activities of 375–540 MBq μmol^−1^. In Kit 3, sodium tartrate and stannous chloride amounts were also reduced. However, further decreasing DP-RGD, to 64 μg in Kit 4, reduced radiochemical yields to 65%.

These kit-based reactions were analysed using reverse phase C_18_ radio-HPLC. A very gradual mobile phase gradient allowed separation and observation of two radioactive products (Fig. 1B, see Experimental section, Method 4 for method details). The first is attributed to *trans*-[^99m^TcO_2_(DP-RGD)_2_]^+^ (52%, retention time (RT) = 41.0 min) and the second is attributed to *cis*-[^99m^TcO_2_(DP-RGD)_2_]^+^ (46%, RT = 44.1). These species exhibit similar chromatographic properties to *trans*-[ReO_2_(DP-RGD)_2_]^+^ (RT = 38.3 min) and *cis*-[ReO_2_(DP-RGD)_2_]^+^ (RT = 42.6 min). The difference in retention times between analogous ^99m^Tc and Re complexes is at least in part due to the configuration of the UV and radioactivity (scintillation) detectors in series. The only other discernable radioactive species (<1%) eluted with the solvent front, and corresponds to either unreacted ^99m^TcO_4_^−^ or ^99m^Tc intermediates bound to other kit-based components (*e.g.* tartrate ligand).

**Fig. 2:**
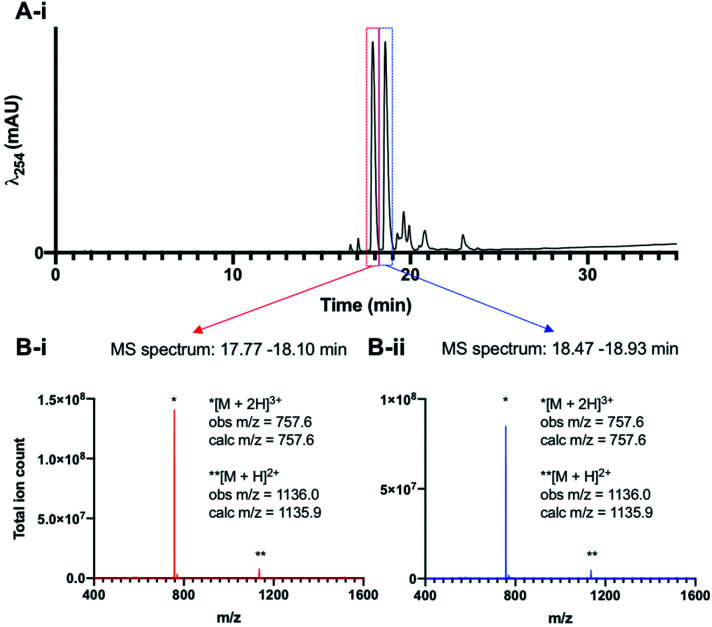
(A) UV (λ_524_) HPLC trace of [^99g^TcO_2_(DP-RGD)_2_]^+^. (B-i and B-ii) The ESI-LRMS corresponding to the two major HPLC signals indicates that the stoichiometry of both of these species corresponds to [^99^TcO_2_(DP-RGD)_2_]^+^ (M = C_110_H_122_N_18_O_22_P_4_^99^Tc^+^).

Lastly, to unambiguously assign the stoichiometry of the [^99m^TcO_2_(DP-RGD)_2_]^+^ compounds, experiments with long-lived technetium-99g (^99g^Tc, *t*_1/2_ = 211 000 years) were undertaken. A sample of [N(C_4_H_9_)_4_][^99g^TcOCl_4_] was reacted with 3 equivalents of DP-RGD in methanol, and analysed by reverse phase C_18_ HPLC methods, which revealed the formation of two major Tc product complexes, with closely similar retention times. LC-ESI-LRMS analysis (Fig. 2) of [^99g^TcO_2_(DP-RGD)_2_]^+^ showed that these two major species possessed LRMS signals consistent with the stoichiometry of [^99^TcO_2_(DP-RGD)_2_]^+^ (*m*/*z* = 757.6 and 1136.0, corresponding to the ions [M + 2H]^3+^ and [M + H]^2+^ where M = [^99^TcO_2_(DP-RGD)_2_]^+^). Additionally, these two major products, tentatively assigned as *trans*-[^99g^TcO_2_(DP-RGD)_2_]^+^ and *cis*-[^99g^TcO_2_(DP-RGD)_2_]^+^ co-eluted with radioactive signals of [^99m^TcO_2_(DP-RGD)_2_]^+^ (Fig. S8†).

### Biological characterisation of [^99m^TcO_2_(DP-RGD)_2_]^+^

For all subsequent experiments, including *in vivo* experiments, [^99m^TcO_2_(DP-RGD)_2_]^+^ was prepared from generator-produced ^99m^TcO_4_^−^ and “Kit 3”, using our newly established one-step radiolabelling protocol. Solutions containing both the desired *cis*-[^99m^TcO_2_(DP-RGD)_2_]^+^ and *trans*-[^99m^TcO_2_(DP-RGD)_2_]^+^ products, as well as unreacted DP-RGD ligand, were used without further purification.

**Fig. 3:**
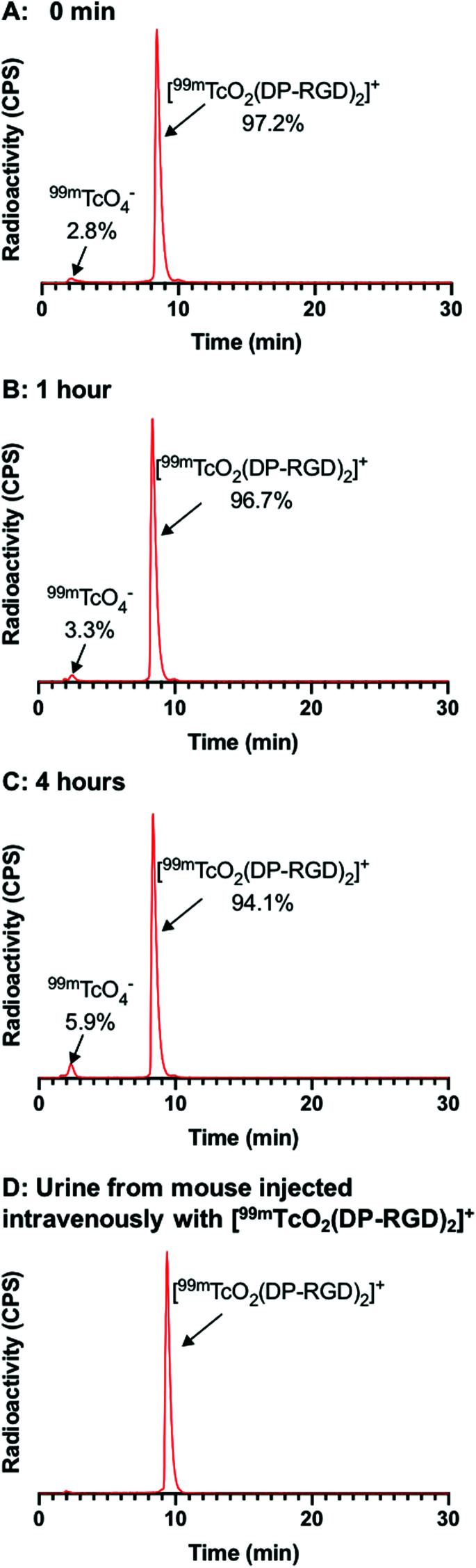
(A–C) [^99m^TcO_2_(DP-RGD)_2_]^+^ was incubated in human serum for 4 h. Analytical radio-HPLC analysis showed that 0.5% ^99m^Tc dissociated from [^99m^TcO_2_(DP-RGD)_2_]^+^ over 1 h, and 3% ^99m^Tc dissociated from [^99m^TcO_2_(DP-RGD)_2_]^+^ over 4 h. (D) Radio-HPLC analysis of urine, collected from a mouse administered [^99m^TcO_2_(DP-RGD)_2_]^+^, showed that [^99m^TcO_2_(DP-RGD)_2_]^+^ was excreted intact. N.B. In these experiments, a short HPLC method, with a relatively steep mobile phase gradient was used: under these conditions, *cis*-[^99m^TcO_2_(DP-RGD)_2_]^+^ and *trans*-[^99m^TcO_2_(DP-RGD)_2_]^+^ were not separated from one another, and eluted as a single peak.

**Fig. 4:**
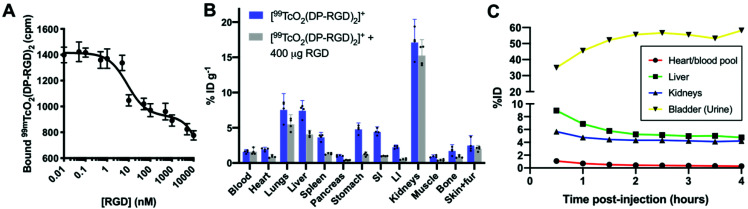
(A) [^99m^TcO_2_(DP-RGD)_2_]^+^ exhibits binding to α_v_β_3_ integrin receptor, which can be inhibited by increasing concentrations of RGD peptide. (B) Biodistribution of [^99m^TcO_2_(DP-RGD)_2_]^+^ in healthy mice 1 h PI: co-injection of 400 μg RGD inhibits [^99m^TcO_2_(DP-RGD)_2_]^+^ uptake in α_v_β_3_ integrin-expressing tissue. Error bars correspond to 95% confidence interval. (C) Quantification of radioactivity distribution from SPECT/CT imaging (Fig. S9†) of a single healthy Balb/c mouse administered [^99m^TcO_2_(DP-RGD)_2_]^+^ intravenously.

The stability of [^99m^TcO_2_(DP-RGD)_2_]^+^ was assessed, by incubating [^99m^TcO_2_(DP-RGD)_2_]^+^ in human serum. C_18_ radio-HPLC analysis revealed that only 3% ^99m^Tc dissociated from [^99m^TcO_2_(DP-RGD)_2_]^+^ over 4 h (Fig. 3), presumably forming ^99m^TcO_4_^−^ (*vide infra*). The log *D*_OCT/PBS_ of [^99m^TcO_2_(DP-RGD)_2_]^+^ measured −1.64 ± 0.04. In a cell-free solid phase α_v_β_3_-integrin receptor binding assay,^20^ [^99m^TcO_2_(DP-RGD)_2_]^+^ bound to α_v_β_3_-integrin receptor, with the binding inhibited by RGD peptide in a concentration-dependent manner (Fig. 4A).

To assess biodistribution, a group of healthy Balb/c mice was intravenously administered with [^99m^TcO_2_(DP-RGD)_2_]^+^ (4.3–5.3 MBq). The α_v_β_3_-integrin receptor is known to be expressed at low levels in normal vasculature. A second group of mice was co-administered with [^99m^TcO_2_(DP-RGD)_2_]^+^ (2.7–4.8 MBq) and a large “excess” of RGD peptide (400 μg), to saturate α_v_β_3_-integrin receptors and “block” receptor-mediated radiotracer accumulation. All mice were culled and their organs harvested for *ex vivo* tissue counting, 1 h post injection (PI). Between the two groups of mice there were statistically significant differences in biodistribution: co-administration of RGD peptide significantly decreased ^99m^Tc radioactivity concentration in the heart, liver, spleen, pancreas, muscles, stomach and intestines (Fig. 4B and Table S3†). This is consistent with the known expression pattern of α_v_β_3_-integrin, and evidences the affinity and specificity of [^99m^TcO_2_(DP-RGD)_2_]^+^ for α_v_β_3_-integrin receptors.^20^ [^99m^TcO_2_(DP-RGD)_2_]^+^ was cleared from circulation *via* a renal pathway, as evidenced by high concentrations of ^99m^Tc in kidneys. Quantitative SPECT/CT image analysis of [^99m^TcO_2_(DP-RGD)_2_]^+^ in a healthy mouse (Fig. 4C and S9†) confirmed that [^99m^TcO_2_(DP-RGD)_2_]^+^ was indeed excreted renally: at 30 min PI, 35% of the injected radioactivity was in the bladder; at 2 h PI, 56% was in the bladder. This was consistent with *ex vivo* tissue counting data. Notably, radio-HPLC analysis of urine showed that [^99m^TcO_2_(DP-RGD)_2_]^+^ was excreted intact (Fig. 3D), consistent with the observed high serum stability.

**Fig. 5:**
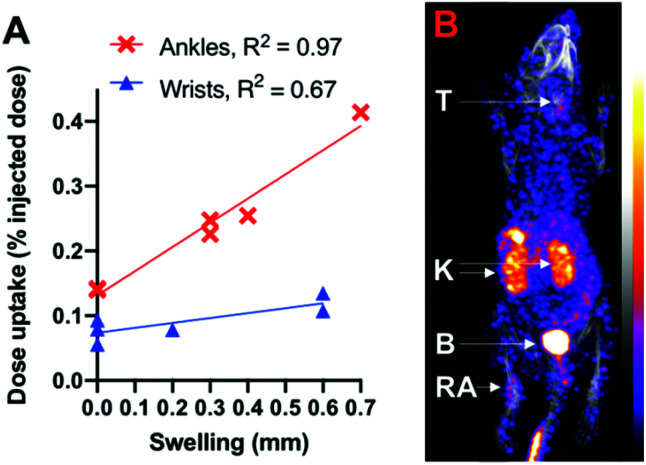
(A) In mice with rheumatoid arthritis, radioactivity accumulation in ankles and wrists correlates with joint swelling. (B) Maximum intensity projection of a SPECT/CT image of a mouse with rheumatoid arthritis, showing accumulation of [^99m^TcO_2_(DP-RGD)_2_]^+^ in an arthritic ankle (RA). B = bladder, K = kidneys, T = thyroid.

Finally, to demonstrate that this new radiotracer can image α_v_β_3_-integrin receptor expression in disease, [^99m^TcO_2_(DP-RGD)_2_]^+^ (4.3–5.2 MBq) was administered to mice induced with rheumatoid arthritis (RA), in which the expression of α_v_β_3_-integrin receptor is associated with inflammation.^20,25^ This is a “heterogeneous” RA model: the degree of arthritis and symptomatic swelling differs between mice, and even between joints of the same animal.^20,26^ Quantitative analysis (Fig. 5A and Fig. S10†) of SPECT images (*e.g.*Fig. 5B) obtained 1 h PI revealed that ^99m^Tc radioactivity accumulation and concentration in wrists and ankles correlated with the degree of symptomatic arthritic swelling, measured with calipers. ^99m^Tc activity was also observed in thyroid tissue: ^99m^TcO_4_^−^ acts as an iodide “mimic” *in vivo*, and is well-documented to be a substrate for the sodium iodide symporter expressed in the thyroid.^27^ It is likely that the observation of ^99m^Tc activity in the thyroid is a result of small amounts of ^99m^Tc dissociating from [^99m^TcO_2_(DP-RGD)_2_]^+^ and forming ^99m^TcO_4_^−^, consistent with the serum stability data.

## Concluding remarks

New chemical platforms that enable one-step, kit-based ^99m^Tc-radiolabelling of peptides could allow increased and widespread clinical use of receptor-targeted ^99m^Tc radiopharmaceuticals. We have shown that BMA is an excellent candidate for this purpose: its conjugates are simple to prepare and can be easily labelled with ^99m^Tc using one-step, kit-based protocols. The biological properties of its peptide conjugates are favourable, as exemplified by [^99m^TcO_2_(DP-RGD)_2_]^+^.

Phosphines have previously been successfully incorporated into chelator–peptide bioconjugates for ^99m^Tc binding.^28–32^ This includes derivatives of a tetradentate P_2_S_2_ ligand (Chart 1), which contains two tertiary alkyl-substituted phosphine groups, for coordinating [TcO_2_]^+^.^15–17^ Small amounts (<1 μmol) of P_2_S_2_-peptide compounds can be radiolabelled with ^99m^TcO_4_^−^ in the presence of stannous chloride to yield ^99m^Tc-labelled peptides of formula [^99m^TcO_2_(P_2_S_2_-peptide)]^+^, in high radiochemical yields (80–98%),^15^ similar to the radiochemical yields we achieve for [^99m^TcO_2_(DP-RGD)_2_]^+^. However, compared to the P_2_S_2_-peptide compounds, bioconjugates of BMA are more synthetically accessible. BMA itself is prepared in two steps^21,22^ from readily available starting materials, and the diphosphine-peptide derivatives of BMA are prepared simply by addition of a peptide containing a single primary amine group to BMA in the presence of base, followed by reverse-phase chromatographic purification.

^99m^Tc-labelled diphosphine-peptide radiotracers based on BMA have advantages over existing ^99m^Tc receptor-targeted radiopharmaceuticals. The radiochemical synthesis of [^99m^TcO_2_(DP-RGD)_2_]^+^ is achieved in a single step, from a single vial, in radiochemical yields >90%. This contrasts with existing molecular ^99m^Tc radiotracers that have recently entered late-stage clinical trials, such as ^99m^Tc-MIP-1404, which requires (i) radiochemical synthesis of *fac*-[^99m^Tc(CO)_3_(H_2_O)_3_]^+^, prior to (ii) reaction with the tridentate MIP-1404 chelator–peptide, and (iii) further purification and formulation before administration.^6^ Additionally, the coordination sphere of [^99m^TcO_2_(DP-RGD)_2_]^+^ is structurally well-defined; the structures of other recently described ^99m^Tc radiopharmaceuticals based on the HYNIC chelator (such as ^99m^Tc-3PRGD2^9^ and ^99m^Tc-HYNIC-PSMA,^10^ which have both demonstrated clinical utility), are more ambiguous.^11,12^ We are currently developing new diphosphine-peptide bioconjugates based on BMA, and further optimising kit-based ^99m^Tc-radiolabelling of such derivatives.

The presence of two isomeric radiolabelled products for DP-peptide conjugates, *cis*-[^99m^TcO_2_(DP-peptide)_2_]^+^ and *trans*-[^99m^TcO_2_(DP-peptide)_2_]^+^, is potentially disadvantageous. It is possible that prior to any clinical application, *cis* and *trans* isomers would require separate biological evaluation, to assess whether their target affinities, pharmacokinetics and stabilities are equivalent to each other. Notably, the ^68^Ga-labelled prostate cancer radiotracer, ^68^Ga-HBED-PSMA, consists of at least two distinguishable (and as yet, undefined) chemical species.^33,34^ However, the biological profiles of each separate ^68^Ga-HBED-PSMA species have not been elucidated, and this has not prevented widespread and routine use of ^68^Ga-HBED-PSMA, and its recent FDA approval, for clinical prostate cancer imaging.^4,35^

Lastly, ^99m^Tc-radiolabelled peptide derivatives of BMA possess a significant advantage over existing receptor-targeted ^99m^Tc radiotracers: upon radiolabelling with the [TcO_2_]^+^ motif, there are two copies of the peptide per molecule. Radiotracers containing two or more peptide copies typically demonstrate higher tumour uptake compared to their monomeric homologues, due to their higher affinity for target receptors.^9,20,36–40^ These examples include ^99m^Tc-labelled compounds that incorporate two copies of a targeting peptide into a single coordinating ligand.^9,38^ However there are only a handful of examples in which ^99m^Tc coordination by two or more copies of a ligand results in formation of radiotracers containing multiple copies of a targeting motif.^41–43^ We have shown that it is feasible to apply diphosphine-peptide bioconjugates for this purpose.

## Experimental methods

### General

All chemicals were supplied by Sigma-Aldrich or Fisher Scientific if not otherwise specified. Sodium [^99m/99^Tc]pertechnetate in saline was supplied by Guy's and St Thomas’ Hospital Nuclear Medicine Services. Cyclic **RGD** peptide (Arg-Gly-Asp-D-Phe-Lys, cyclised *via* the peptide backbone) was purchased from Peptide Synthetics (Hampshire, UK).

NMR data (^1^H, ^13^C{H} and ^31^P{H} 1D spectra and COSY, TOCSY and HSQC spectra) were acquired on a Bruker Avance III 400 spectrometer equipped with a QNP probe or a Bruker Avance III 700 spectrometer equipped with an AVIII console and a quadruple-resonance QCI cryoprobe. High resolution mass spectrometry (MS) was performed by the King's College London Mass Spectrometry Facilities, using a high resolution Thermo Exactive mass spectrometer in positive electrospray mode. Samples were infused to the ion source at a rate of 10 μl min^−1^ using a syringe pump. High performance liquid chromatography (HPLC) was carried out on an Agilent 1200 LC system with the Laura software, a Rheodyne sample loop (200 μL) and UV spectroscopic detection at 220 nm or 254 nm. The HPLC was attached to a LabLogic Flow-Count detector with a sodium iodide probe (B-FC-3200) for radiation detection. LC-ESI-LRMS was carried out on an Agilent 1260 Infinity II HPLC system coupled to an Advion Expression Compact Mass Spectrometer using an ESI source. Semi-preparative (9.4 × 250 mm, 5 μm) and analytical (4.6 × 150 mm, 5 μm) Agilent Zorbax Eclipse XDB-C18 columns were used with purified water (A) and acetonitrile (B) containing 0.005% and 0.1% TFA as mobile phases for semi-preparative and analytical runs, respectively. **Method 1** (semi-preparative): 100 minutes, 1% min^−1^ linear increase from 100% A to 100% B, flow rate = 3 mL min^−1^. **Method 2** (analytical): 20 minutes, 5% min^−1^ linear increase from 100% A to 100% B, flow rate = 1 mL min^−1^. **Method 3** (semi-preparative): 200 minutes, 0.5% min^−1^ linear increase from 95% A to 100% B, flow rate = 3 mL min^−1^. **Method 4** (analytical): 55 minutes, 2.5% min^−1^ linear increase from 100% A to 75%A/25% B over 10 min, followed by 0.33% min^−1^ linear increase from 75% A/25% B to 60%A/40% B over 45 min, flow rate of 1 mL min^−1^. **Method 5** (analytical, 0.1% formic acid in either water (A) or acetonitrile (B)): 0–5 min: 95% A/5% B; 5–35 min: linear increase from 95% A/5% B to 5% A/95% B; flow rate of 1 mL min^−1^.

### Synthesis

#### DP-RGD

Solutions of BMA (prepared as previously described^21,22^) **1** (5.30 mg, 11.40 μmol) in DMF (100 μL) and **RGD** peptide (7.00 mg, 11.00 μmol) in DMF (100 μL) were combined and *N*,*N*-diisopropylethylamine (DIPEA, 6 μL) added. The resulting orange solution was stirred at room temperature for 10 min, yielding a yellow solution. The solution was applied to a semi-preparative HPLC column, and components separated using HPLC method 1. A solution of aqueous ammonium bicarbonate (0.125 M) was added to each fraction containing DP-RGD, at a ratio of 10 μL of ammonium bicarbonate solution:1 mL of HPLC eluate. Solutions containing DP-RGD were lyophilised. DP-RGD yield = 10.10 mg, 9.44 μmol, 86%. Full ^1^H and ^13^C NMR data in ESI.†^31^P NMR (283 MHz, DMF-d_7_, 298 K): *δ* (ppm) −12.98 (d, *J* = 168.1 Hz), −11.95 (d, *J* = 168.1 Hz). HR-MS-ESI *m*/*z*: [M + H]^+^ 1070.4070 (calculated for C_55_H_62_O_10_N_9_P_2_^+^ 1070.4089).

#### *cis*/*trans*-[ReO_2_(DP-RGD)_2_]^+^

A solution of [ReO_2_I(PPh3)_2_] (3.0 mg, 3.45 μmol) in DMF (100 μL) was combined with a solution of DP-RGD (3.7 mg, 3.45 μmol) and DIPEA (6 μL) in DMF (200 μL). The resulting dark brown/black solution was agitated at room temperature for 10 min. Upon addition of ice-cold diethyl ether, a precipitate formed. The supernatant was removed, and the precipitate was dissolved in DMF (200 μL) and applied to a semi-preparative HPLC column. Reaction components were separated using HPLC method 3. A solution of aqueous ammonium bicarbonate (0.125 M) was added to each fraction containing *cis*/*trans*-[ReO_2_(DP-RGD)_2_]^+^ at a ratio of 10 μL of ammonium bicarbonate solution: 1 mL of HPLC eluate. Solutions containing *cis*/*trans*-[ReO_2_(DP-RGD)_2_]^+^ were lyophilised. The lyophilised fractions that eluted at 65–67 min and 68–70 min were identified as *trans*-[ReO_2_(DPP-N-RGD)_2_]^+^ 0.8 mg, 0.34 μmol, 9.9% and *cis*-[ReO_2_(DPP-N-RGD)_2_]^+^ (0.9 mg, 0.38 μmol, 11.0%), respectively. Full ^1^H and ^13^C NMR data in ESI.†***trans*-[ReO**_**2**_**(DP-RGD)**_**2**_**]**^**+**^: ^31^P NMR (283 MHz, DMF-d_7_, 298 K): *δ* (ppm) 23.781 (m), 24.506 (m). HR-MS-ESI *m*/*z*: [M + H]^2+^ 1179.3826 (calculated for C_110_H_123_N_18_O_22_P_4_Re^2+^ 1179.3773), [M + 2H]^3+^ 786.5921 (calculated for C_110_H_124_N_18_O_22_P_4_Re^3+^ 786.5906). ***cis*-[ReO**_**2**_**(DP-RGD)**_**2**_**]**^**+**^: ^31^P NMR (283 MHz, DMF-d_7_, 298 K): *δ* (ppm) 21.848 (dm, *J*_1_ = 356.1 Hz), 26.335 (dm, *J*_1_ = 356.1 Hz). HRMS-ESI *m*/*z*: [M + H]^2+^ 1179.3826 (calculated for C_110_H_123_N_18_O_22_P_4_Re^2+^ 1179.3773), [M + 2H]^3+^ 786.5921 (calculated for C_110_H_124_N_18_O_22_P_4_Re^3+^ 786.5906).

### Preparation and characterisation of [^99m/99g^TcO_2_(DP-RGD)_2_]^+^

#### Radiolabelling kits

An aqueous stock solution was prepared containing the required amounts of sodium bicarbonate, tin chloride dihydrate and either sodium gluconate or sodium tartrate dibasic dihydrate. The pH of this solution was adjusted to 8.5 by dropwise addition of an aqueous solution of sodium hydroxide (0.1 M). Aliquots of the stock solution were mixed with the required amount of DP-RGD (in ethanol), and the resulting solutions (Table 1) were frozen and lyophilised. The lyophilised kits were stored at −18 °C prior to use.

#### ^99m^Tc radiolabelling

DP-RGD was radiolabelled with generator-produced ^99m^TcO_4_^−^ in saline solution (0.9% NaCl in water, w/v). Saline solution containing ^99m^TcO_4_^−^ and ethanol were added to the contents of a “kit” (amounts listed in Table 1). The radiolabelling kit mixture was heated at 60 °C for 30 min, and then analysed by analytical HPLC (method 2) and instant thin layer chromatography (iTLC) using iTLC SGI0001 strips (9 or 10 cm length; Varian Medical Systems, Crawley, UK). The iTLC plates were scanned with a PerkinElmer Storage Phosphor System (Cyclone) or a LabLogic miniScan TLC reader equipped with Laura software.

Two separate iTLC analyses were undertaken, to enable quantification of ^99m^Tc-colloids, unreacted ^99m^TcO_4_^−^ and [^99m^TcO_2_(DP-RGD)_2_]^+^. To quantify amounts of unreacted ^99m^TcO_4_^−^, acetone was used as a mobile phase: *R*_f_ values: ^99m^TcO_4_^−^ >0.9, ^99m^Tc colloids <0.1, [^99m^TcO_2_(DP-RGD)_2_]^+^ <0.1. To quantify ^99m^Tc-colloid formation, a 1 : 1 mixture of methanol and 2 M aqueous ammonium acetate solution was used as a mobile phase: ^99m^TcO_4_^−^ >0.9, ^99m^Tc colloids <0.1, [^99m^TcO_2_(DP-RGD)_2_]^+^ >0.9.

#### ^99g^Tc radiolabelling

[N(C_4_H_9_)_4_][^99g^TcOCl_4_]^44^ (0.4 mg) dissolved in methanol (50 μL) was added to DP-RGD (2.4 mg, 3 equiv.) dissolved in methanol (200 μL), resulting in a yellow-orange solution. The sample was analysed by reverse-phase analytical HPLC (method 4, *vide infra*), and LC-ESI-LRMS (method 5). ESI-LRMS *m*/*z*: [M + H]^2+^ 1136.0 (calculated for C_110_H_123_N_18_O_22_P_4_^99^Tc^2+^ 1135.9), [M + 2H]^3+^ 757.6 (calculated for C_110_H_124_N_18_O_22_P_4_^99^Tc^3+^ 757.6).

#### Co-elution of [^99m^TcO_2_(DP-RGD)_2_]^+^ with *cis*/*trans*-[ReO_2_(DP-RGD)_2_]^+^ and *cis*/*trans*-[^99g^TcO_2_(DP-RGD)_2_]^+^

[^99m^TcO_2_(DP-RGD)_2_]^+^ was prepared in >90% RCY as described above, and co-injected with *cis*-[ReO_2_(DP-RGD)_2_]^+^ and separately, *trans*-[ReO_2_(DP-RGD)_2_]^+^, onto a reverse-phase analytical HPLC column (method 4). A sample of *cis*/*trans*-[^99g^TcO_2_(DP-RGD)_2_]^+^ was also analysed, using the same HPLC method. Retention times: *trans*/*cis*-[^99m^TcO_2_(DP-RGD)_2_]^+^ 41.0 min and 44.1 min (NaI scintillator detection); *trans*/*cis*-[^99g^TcO_2_(DP-RGD)_2_]^+^ 40.70 min and 43.1 min; *trans*-[ReO_2_(DP-RGD)_2_]^+^ 38.3 min and *cis*-[ReO_2_(DP-RGD)_2_]^+^ 42.6 min.

#### Log *D* (pH 7.4)

The following procedure was carried out in triplicate. A solution containing [^99m^TcO_2_(DP-RGD)_2_]^+^ (1 MBq in 7.5 μL) was combined with phosphate buffered saline (pH 7.4, 500 μL) and octanol (500 μL), and the mixture was agitated for 30 min. The mixture was then centrifuged (10 000 rpm, 10 minutes), and aliquots of octanol and aqueous PBS were analysed for radioactive using a gamma counter. Log *D* = −1.64 ± 0.04.

#### Serum stability

A solution containing [^99m^TcO_2_(DP-RGD)_2_]^+^ (100 μL, 79 MBq) was added to filtered human serum (Sigma-Aldrich, 900 μL) and incubated at 37 °C for 4 h. At 1 and 4 h, aliquots were taken. Each aliquot (300 μL) was treated with ice-cold acetonitrile (300 μL) to precipitate and remove serum proteins. Acetonitrile in the supernatant was then removed by evaporation under a stream of N_2_ gas (40 °C, 30 min). The final solution was then analysed by reverse-phase analytical HPLC (method 2).

#### α_v_β_3_-integrin solid-phase competitive binding assay

The affinity of [^99m^TcO_2_(DP-RGD)_2_]^+^ for α_v_β_3_ integrin was determined in a solid-phase competitive binding assay.^20^ In brief, wells of a 96 well plate were coated with 150 ng mL^−1^ integrin α_v_β_3_ in 100 μL coating buffer (25 mM Tris HCl pH 7.4, 150 mM NaCl, 1 mM CaCl_2_, 0.5 mM MgCl_2_, and 1 mM MnCl_2_) overnight at 4 °C. Wells were then washed twice in binding buffer (coating buffer plus 0.1% bovine serum albumin (BSA)) before being blocked for 2 hours at room temperature with blocking buffer (coating buffer plus 1% BSA). After a further two washes in binding buffer, both [^99m^TcO_2_(DP-RGD)_2_]^+^ (RCY > 96%, 1–2 kBq in 50 μL binding buffer, containing 1.2 pmol DP-RGD peptide) and RGD peptide (10.0 pM to 10 000 nM, 50 μL in binding buffer) were added simultaneously to wells, and left to incubate for 1 h at room temperature, before being washed twice as before. Finally, the amount of activity bound to the wells was counted. Binding of [^99m^TcO_2_(DP-RGD)_2_]^+^ to α_v_β_3_ integrin was displaced by RGD peptide in a concentration-dependent manner. The pseudo-IC_50_ value of 8.54 ± 3.45 nM (95% CI: 1.67–15.41 nM) was calculated using a non-linear regression model (Binding/Saturation, one site – total) in GraphPad Prism (*n* = 6 from one experiment).

### Pre-clinical imaging and *in vivo* biodistribution studies of [^99m^TcO_2_(DP-RGD)_2_]^+^

Animal imaging studies were ethically reviewed and carried out in accordance with the Animals (Scientific Procedures) Act 1986 (ASPA) UK Home Office regulations governing animal experimentation. SPECT/CT imaging was accomplished using a pre-clinical nanoScan SPECT/CT Silver Upgrade instrument (Mediso) calibrated for technetium-99m. All scans were acquired by helical SPECT (4-head scanner with 4 × 9 [1.4 mm] pinhole collimators), and helical CT with 1.4 mm aperture collimators. All acquired images were reconstructed using a full 3D Monte Carlo-based iterative algorithm (Tera-Tomo; Mediso) and further processed and analysed using VivoQuant software (inviCRO, USA).

#### SPECT/CT imaging and biodistribution in healthy mice

A female, balb/c mouse (2 months old) was anaesthetised (2–3% v/v isofluorane in oxygen), scanned by CT and injected intravenously (tail vein) with [^99m^TcO_2_(DP-RGD)_2_]^+^ (21 MBq containing 22 μg of DP-RGD peptide). SPECT images (8 × 30 min images) were acquired over 4 h. At the end of the imaging procedure, the mouse was culled by cervical dislocation and a sample of the urine analysed by reverse-phase HPLC (analytical, method 2).

Female balb/c mice (2 months old) were anaesthetised (2–3% v/v isofluorane in oxygen) and injected intravenously (tail vein) with [^99m^TcO_2_(DP-RGD)_2_]^+^ (2.7–5.3 MBq containing 5 μg of DP-RGD). For blocking studies, animals were co-injected with RGD peptide (400 μg). Mice remained under anaesthetic for 1 h, after which they were culled (pentabarbitone by i.v. injection). Tissues and organs were harvested and weighed, and radioactivity counted using a Gamma Counter (Wallac 1282 CompuGamma Universal Gamma Counter).

#### SPECT/CT imaging and biodistribution in mice induced with rheumatoid arthritis

We used a K/BxN serum transfer arthritis (STA) model of rheumatoid arthritis.^20,26^ On day 0 and 2, female C57Bl/6J mice (2 months old) were injected intraperitoneally with arthritogenic serum in sterile filtered PBS (150 μL, 50% v/v, serum obtained from arthritic K/B × N transgenic mice). Disease severity was evaluated in mice throughout the induction period, by measuring weight, thickness of swollen paws using microcallipers, and visual scoring on a scale of 0–3 per paw. SPECT/CT imaging and biodistribution was undertaken on day 7.

Mice were anesthetised (2.5–3% v/v isofluorane) and their paws were measured using microcallipers. Mice were then injected intravenously with [^99m^TcO_2_(DP-RGD)_2_]^+^ (approx. 5 MBq containing 5 μg of DP-RGD) and allowed to recover from anaesthetic administration. At 1 h post-injection of radiotracer, mice were culled (sodium pentabarbitone), and underwent SPECT/CT scanning post-mortem for 60–180 min. Finally, tissues and organs were harvested and weighed, and radioactivity counted using a Gamma Counter (Wallac 1282 CompuGamma Universal Gamma Counter). The acquired images were processed to units of %ID and the regions of interest (ROIs) delineated by CT using VivoQuant software (inviCRO, USA). Radioactivity in ankle and wrist ROIs were obtained in units of %ID and %ID/cm^−3^. Each “ankle” ROI was defined as the area between the tibiofibula joint and the base of phalanx V. Each “wrist” ROI was defined as the area between the narrowest point of the wrist (ulna and radius) and the end of the forepaw.

## Conflicts of interest

There are no conflicts to declare.

## Supplementary Material

DT-050-D1DT03177E-s001
