# Supplementary material for: One-step, kit-based radiopharmaceuticals for molecular SPECT imaging: a versatile diphosphine chelator for 99mTc radiolabelling of peptides
Source: Dalton Trans. 2021 Oct 15;50(44):16156–65. doi: 10.1039/d1dt03177e (PMC8594432; doi:10.1039/d1dt03177e)
Supplement: DT-050-D1DT03177E-s001 [file DT-050-D1DT03177E-s001.pdf]

Electronic Supplementary Material (ESI) for Dalton Transactions.
This journal is © The Royal Society of Chemistry 2021

One-step, kit-based radiopharmaceuticals for molecular SPECT imaging: a versatile diphosphine chelator for ^{99m}Tc radiolabelling of peptides

SUPPORTING INFORMATION

Ingebjørg N. Hungnes,[a] Fahad Al-Salemee,[a] Peter J. Gawne,[a] Thomas Eykyn,[a] R. Andrew Atkinson,[b,c] Samantha Y. A. Terry,[a] Fiona Clarke,[d] Philip J. Blower,[a] Paul G. Pringle[e] and Michelle T. Ma[a]*

[a] *King's College London, School of Biomedical Engineering and Imaging Sciences, 4th Floor Lambeth Wing, St Thomas' Hospital, London, UK.*
[b] *King's College London, Randall Centre for Cell and Molecular Biophysics and Centre for Biomolecular Spectroscopy, London, UK.*
[c] *Institut de Pharmacologie et de Biologie Structurale, IPBS, Université de Toulouse, CNRS, Université Paul Sabatier, 31077 Toulouse, France.*
[d] *King's College London, Academic Department of Rheumatology, Centre for Molecular and Cellular Biology of Inflammation, Faculty of Life Sciences and Medicine, London, UK.*
[e] *University of Bristol, School of Chemistry, Cantock's Close, Bristol, UK*

email for correspondence: michelle.ma@kcl.ac.uk

Figure S1: HR-ESI-MS Page 2
Figure S2: Analytical reverse phase C18 HPLC trace of DP-RGD Page 3
Figure S3, S4: ^{1}H TOCSY spectra Pages 3-4
Figure S5: ^{1}H-^{13}C HSQC spectra Page 4
Figure S6: Full $^{31}P\{H\}$ NMR of DP-RGD and *trans-/cis-*$[ReO_2(DP\text{-}RGD)_2]^+$ Page 5
Figure S7: Full radio-HPLC trace of *trans-/cis-*$[MO_2(DP\text{-}RGD)_2]^+$ (M = Tc, Re) Page 5
Figure S8: Full radio-HPLC trace of *trans-/cis-*$[^{99m/99g}TcO_2(DP\text{-}RGD)_2]^+$ Page 6
Figure S9: SPECT/CT imaging data of $[^{99m}TcO_2(DP\text{-}RGD)_2]^+$ in a healthy mouse Page 6
Figure S10: Radioactivity concentration in ankles and wrists of rheumatoid arthritic mice Page 7
Figure S11: Biodistribution of $[^{99m}TcO_2(DP\text{-}RGD)_2]^+$ in rheumatoid arthritis mice Page 7

Table S1: Summary of ^{1}H chemical shifts Page 8
Table S2: Summary of ^{13}C chemical shifts Page 9
Table S3: Biodistribution of ^{99m}Tc in healthy balb/c mice Page 10

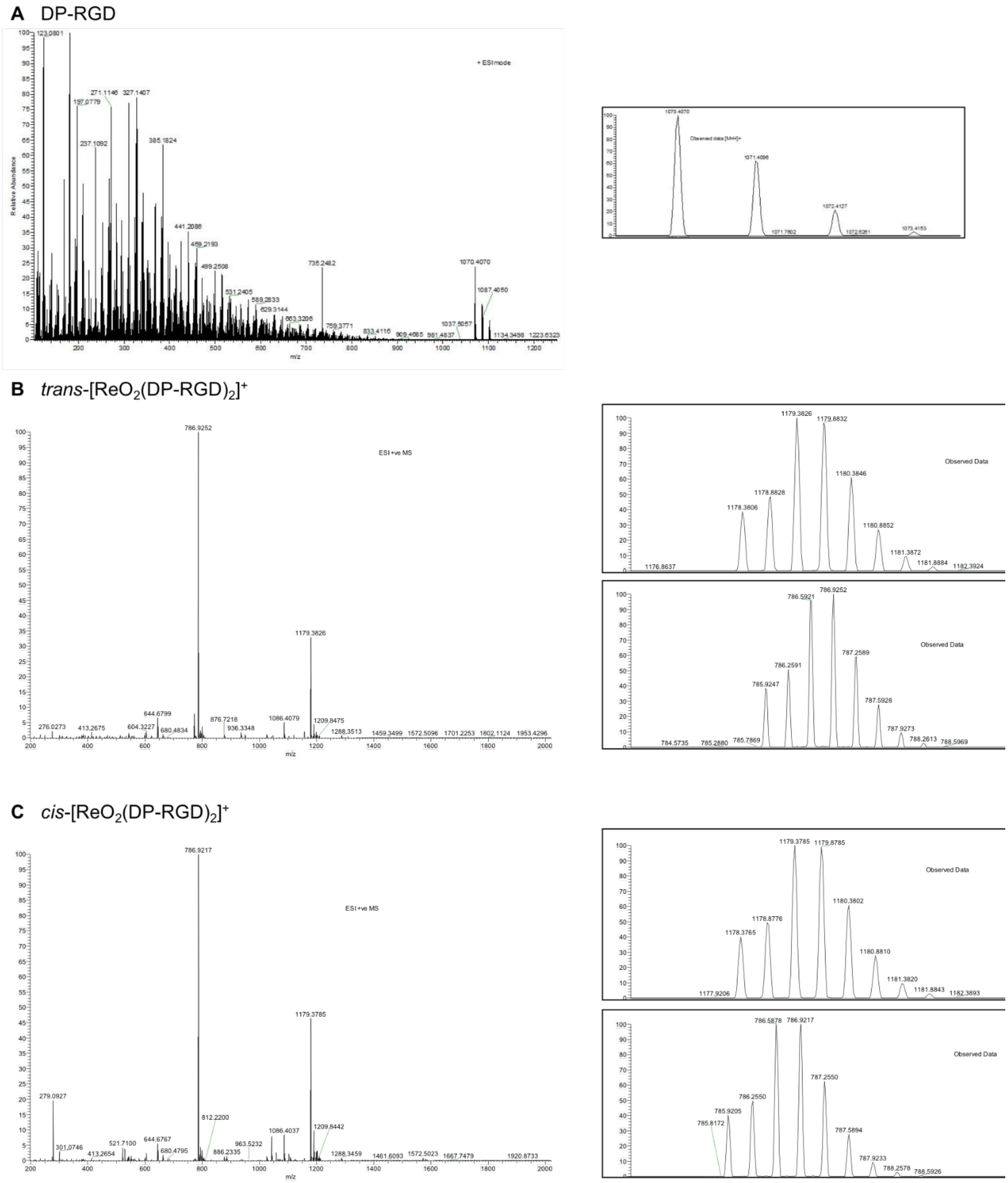

Figure S1 HR-ESI-MS of **(A)** DP-RGD, **(B)** *trans*-$[ReO_2(DP\text{-}RGD)_2]^+$ and **(C)** *cis*-$[ReO_2(DP\text{-}RGD)_2]^+$. For DP-RGD, calculated monoisotopic peak for $[M + H]^+$: $[C_{55}H_{61}O_{10}N_9P_2 + H]^+$, 1070.4089. For both $[ReO_2(DP\text{-}RGD)_2]^+$ species, the major signals in the ESI-MS correspond to the tripositive and dipositive cations of the $[ReO_2(DP\text{-}RGD)_2]^+$ complex. Calculated monoisotopic peak for $[M + 2H]^{3+}$: $[ReC_{110}H_{124}N_{18}O_{22}P_4]^{3+}$, 785.92. Calculated monoisotopic peak for $[M + H]^{2+}$: $[ReC_{110}H_{123}N_{18}O_{22}P_4]^{2+}$, 1178.38.

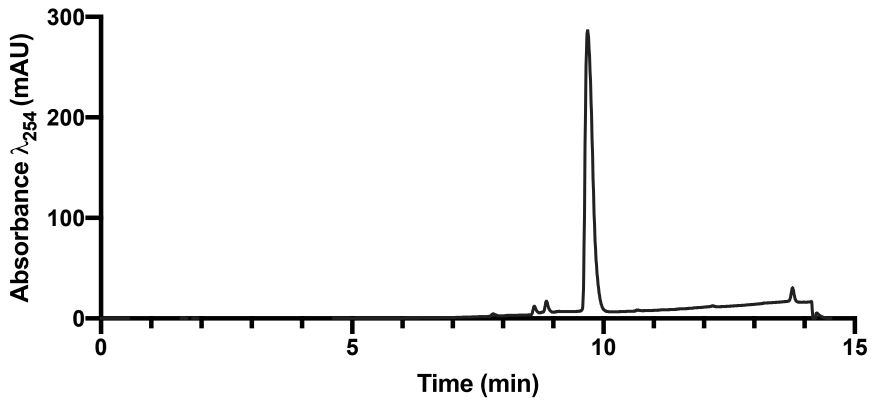

Figure S2 Analytical reverse phase C18 UV (254 nm) HPLC trace of DP-RGD.

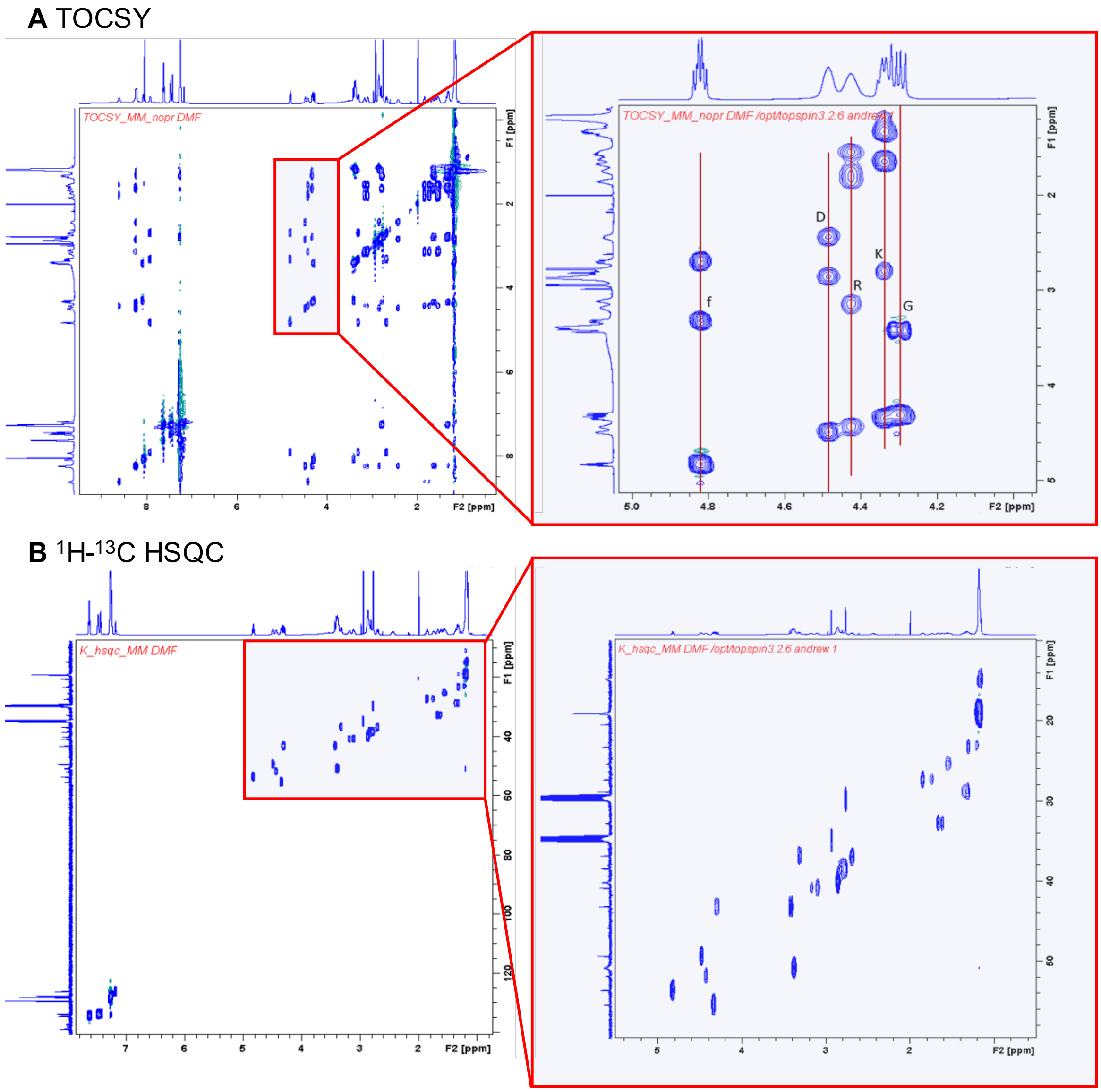

Figure S3 (A) ^{1}H TOCSY spectrum of DP-RGD (left) with expansion (right) showing crosspeak signals from peptide αCH protons and aliphatic protons from their respective amino acid sidechains; **(B)** ^{1}H-^{13}C HSQC spectrum of DP-RGD (left) with expansion (right) of aliphatic region, showing signals from amino acid sidechains.

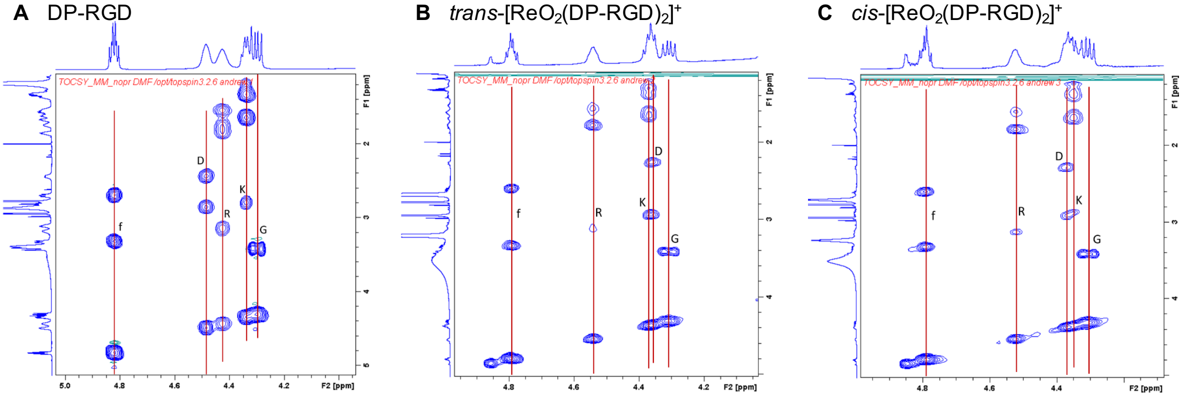

Figure S4 ^{1}H TOCSY spectral regions depicting signals from peptide αCH protons and aliphatic protons from their respective amino acid sidechains, for **(A)** DP-RGD, **(B)** *trans*-$[ReO_2(DP\text{-}RGD)_2]^+$, and **(C)** *cis*-$[ReO_2(DP\text{-}RGD)_2]^+$. With the exception of aspartic acid (D) chemical shifts, which are sensitive to changes in solvent pH, ^{1}H chemical shifts for the RGD peptide motif are minimally affected by Re^V binding.

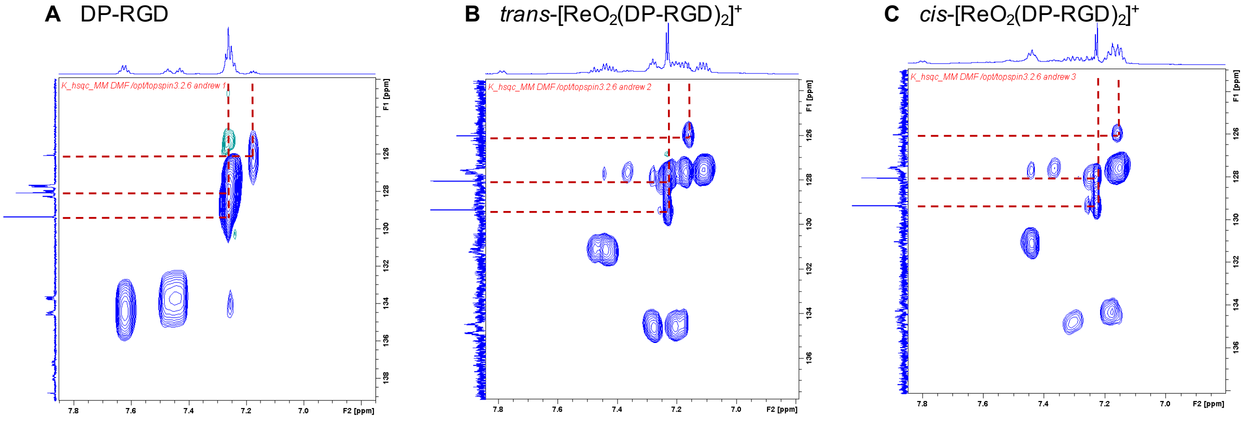

Figure S5 ^{1}H-^{13}C HSQC spectra of aromatic regions, for **(A)** DP-RGD, **(B)** *trans*-$[ReO_2(DP\text{-}RGD)_2]^+$, and **(C)** *cis*-$[ReO_2(DP\text{-}RGD)_2]^+$. The dotted red lines denote signals arising from the D-phenylalanine sidechain, whilst other crosspeak signals arise from the P-C_6H_5 groups. The ^{1}H and ^{13}C chemical shifts arising from the D-phenylalanine residue are minimally affected by Re^V binding. However, HSQC spectra indicated subtle changes in both the ^{1}H and ^{13}C chemical shifts of P-C_6H_5 groups upon Re^V binding. This includes an increase in the complexity of the ^{1}H spectral region.

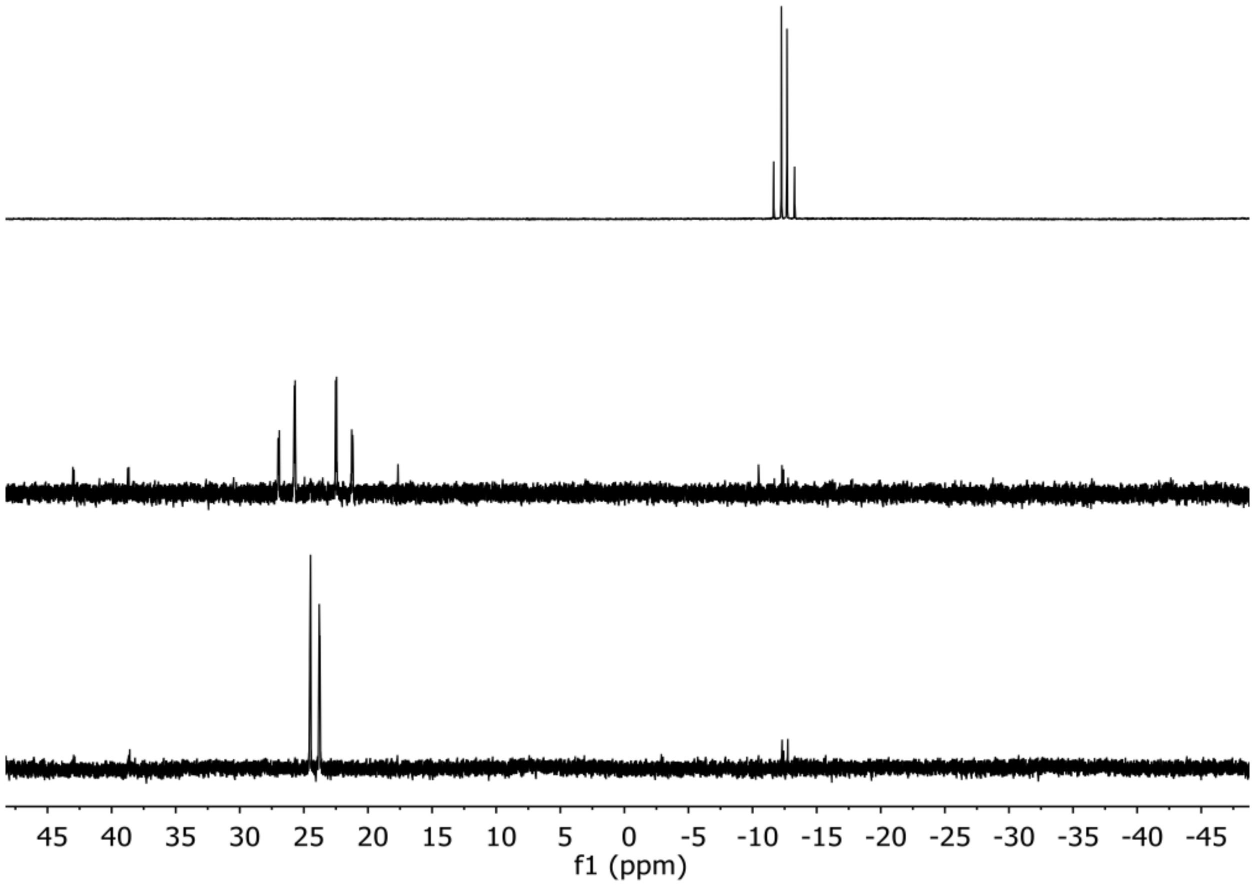

Figure S6 Full ³¹P{H} NMR of DP-RGD (top), *cis*-$[Re(O)_2(DP\text{-}RGD)_2]^+$ (middle) and *trans*-$[Re(O)_2(DP\text{-}RGD)_2]^+$ (bottom).

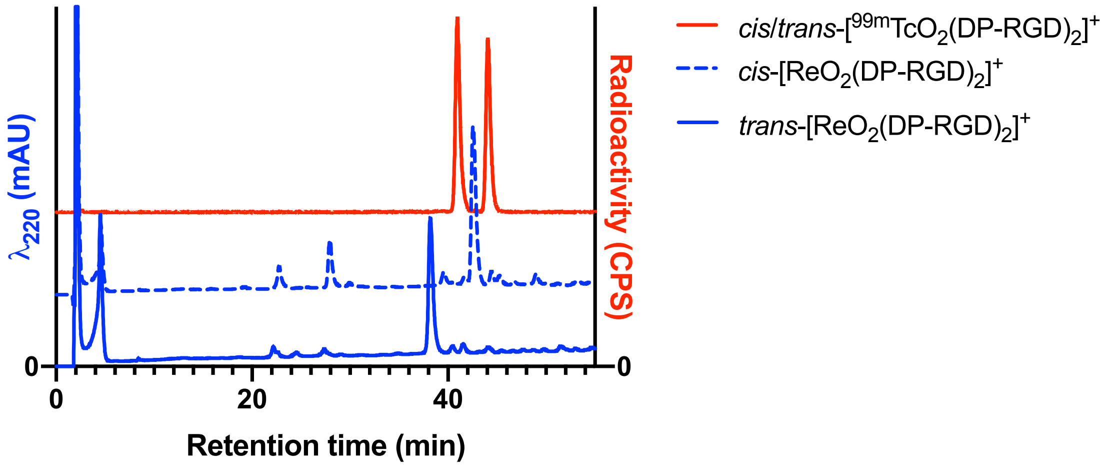

Figure S7 Radio-HPLC trace of *trans-/cis*-$[^{99m}TcO_2(DP\text{-}RGD)_2]^+$ (red) prepared from an aqueous solution of $^{99m}TcO_4^-$ and Kit 3, and HPLC traces (λ_{220}) of *trans*- and *cis*-$[ReO_2(DP\text{-}RGD)_2]^+$ (blue). The difference in retention times between analogous ^{99m}Tc and Re complexes is at least in part due to the configuration of the UV and scintillation detectors in series.

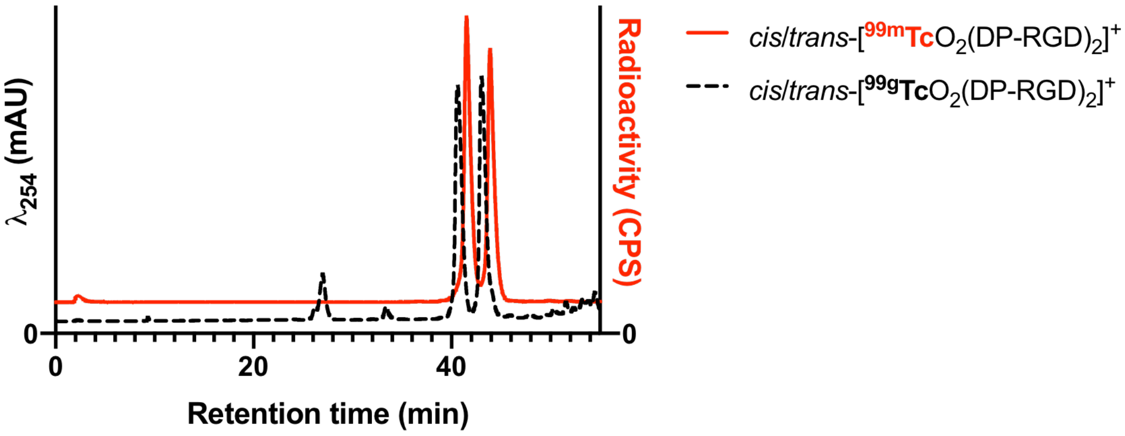

Figure S8 Radio-HPLC trace of *trans-/cis-*[^{99m}TcO$_2$(DP-RGD)$_2$]$^+$ (red) and UV-HPLC trace of *trans-/cis-*[^{99g}TcO$_2$(DP-RGD)$_2$]$^+$ (black). The difference in retention times between ^{99m}Tc and ^{99g}Tc isotopologues is due to the configuration of the UV and scintillation detectors in series.

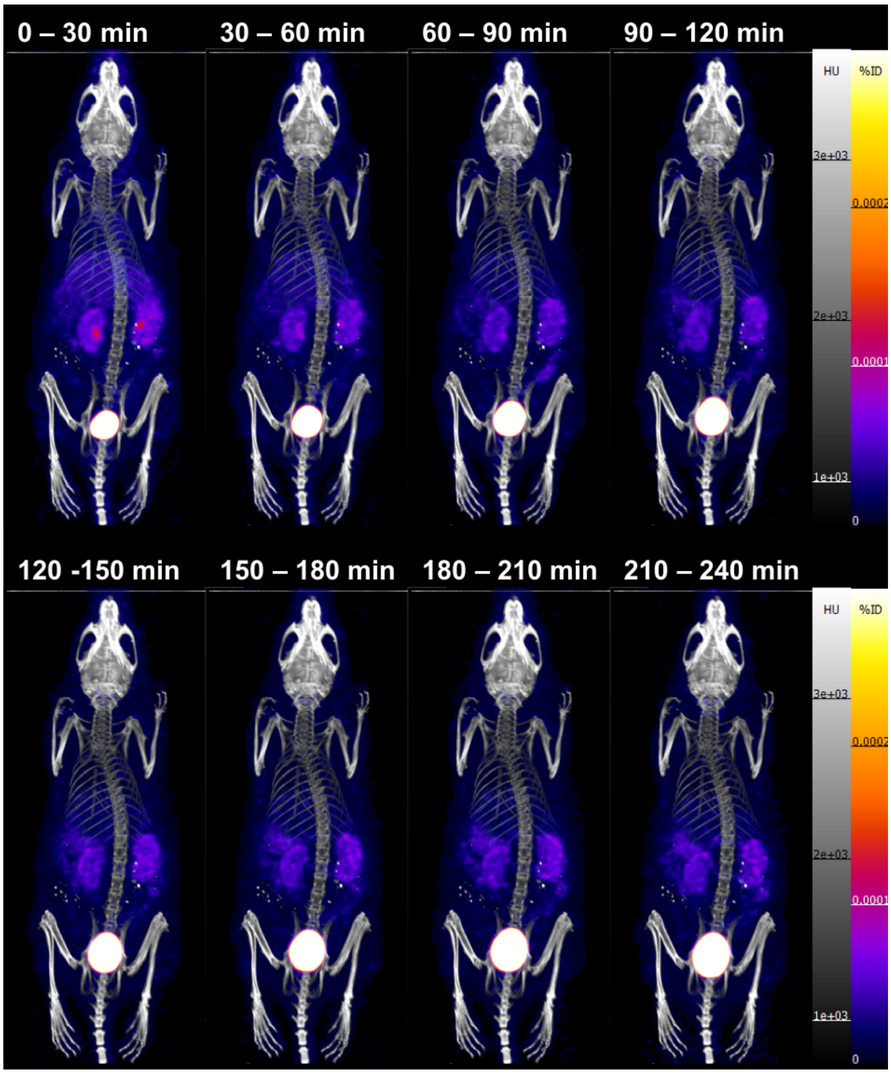

Figure S9 SPECT/CT maximum intensity projection images of a balb/c mouse administered [^{99m}TcO$_2$(DP-RGD)$_2$]$^+$ intravenously. SPECT images were acquired over 4 hours in 30 min segments. Imaging analysis indicated that the majority of [^{99m}TcO$_2$(DP-RGD)$_2$]$^+$ cleared rapidly via a renal pathway: at 30 min PI (post-injection), 35% of the injected dose of radioactivity was in the bladder; at 2 h PI, 56% was in the bladder.

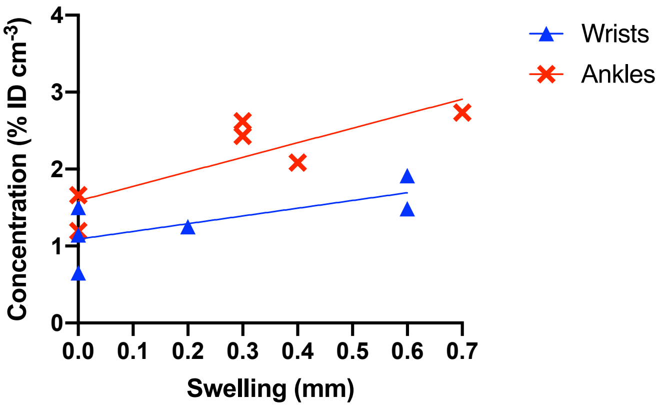

Figure S10 In mice with induced rheumatoid arthritis, radioactivity concentration in ankles and wrists (measured using SPECT/CT image quantification) correlates with degree of joint swelling (measured using calipers). For ankles, y = 1.89x + 1.587, R^2 = 0.69, and p = 0.04 (significance of slope from non-zero). For wrists, y = 1.01*x + 1.09, R^2 = 0.49, and p = 0.12.

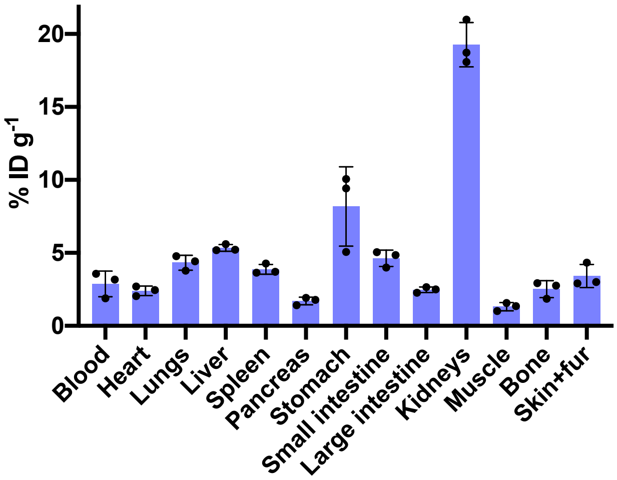

Figure S11 Biodistribution of $[^{99m}TcO_2(DP\text{-}RGD)_2]^+$ in rheumatoid arthritis mice 1 h post-injection (n = 3). Error bars correspond to standard deviation.

Table S1: Summary of ^{1}H chemical shifts

^{1}H NMR		DP-RGD			*trans*-[ReO$_2$(DP-RGD)$_2$]$^+$			*cis*-[ReO$_2$(DP-RGD)$_2$]$^+$		
		δ / ppm		*J* / Hz	*δ* / ppm		*J* / Hz	*δ* / ppm		*J* / Hz
**DP**	CH(d/d′)	7.652	q	7.17	7.281	m		7.305	m	
		7.452	dt	29.19, 6.76	7.204	m		7.175	m	
	CH(e/e′)	7.269	m		7.170	m		7.151	m	
					7.111	m				
	CH(f/f′)	7.269	m		7.477	m		7.439	m	
					7.436	m				
**Lys**	NH	8.237	m		8.196	m		8.253	d	7.46
	CH(α)	4.337	m		4.365	m		4.367	m	
	CH2(β)	1.664	m		1.681	m		1.664	m	
		1.616	m					1.599	m	
	CH2(γ)	1.153	m		1.184	m		1.188	m	
	CH2(δ)	1.334	m		1.300	m		1.300	m	
	CH2(ε)	2.807	m		2.943	-		2.876	m	
		2.771	m							
	NH(ζ)	7.257	-		8.294	-		8.088	m	
**Arg**	NH	8.610	s		8.629	d	8.98	8.621	d	8.85
	CH(α)	4.425	s		4.543	m		4.525	m	
	CH2(β)	1.847	m		1.612	m		1.608	m	
		1.737	s br		1.511	m		1.528	m	
	CH2(γ)	1.546	m		1.769	m		1.785	m	
	CH2(δ)	3.170	s br		3.144	m		3.148	m	
		3.104	m		3.067	m		3.109	m	
**Gly**	NH	8.082	d	7.06	8.089	m		8.087	m	
	CH(α)	4.301	dd	16.29, 9.01	4.307	dd	16.24, 2.67	4.308	dd	16.64, 9.05
		3.413	dd	16.45, 2.98	3.416	dd	16.45, 9.05	3.415	-	
**Asp**	NH	8.237	m		8.358	d	9.32	8.335	d	8.85
	CH(α)	4.484	s		4.358	m		4.379	m	
	CH2(β)	2.428	m		2.940	m		2.924	-	
		2.840	m		2.261	m		2.283	m	
**Phe**	NH	7.792	d	7.68	7.787	d	8.98	7.798	d	9.37
	CH(α)	4.821	td	8.74, 5.84	4.796	m		4.789	m	
	CH2(β)	3.314	dd	14.22, 5.44	3.330	dd	9.26, 5.00	3.319	dd	14.51, 5.14
		2.688	dd	13.80, 8.92	2.601	dd	14.51, 9.26	2.606	m	
	CH(o)	7.269	m		7.232	m		7.220	m	
	CH(m)	7.269	m		7.232	m		7.220	m	
	CH(p)	7.258	m		7.161	m		7.158	m	

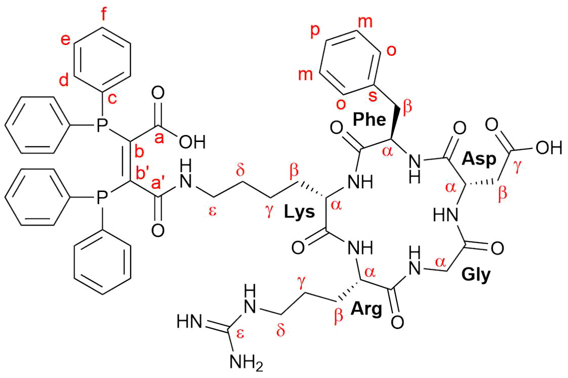

Table S2: Summary of ^{13}C chemical shifts

	^{13}C NMR	DP-RGD		*trans*-[ReO$_2$(DP-RGD)$_2$]$^+$		*cis*-[ReO$_2$(DP-RGD)$_2$]$^+$	
		δ / ppm	mult.	δ / ppm	mult.	δ / ppm	mult.
**DP**	$C_{a/a'}$	*		**		***	
	$C_{b/b'}$	*		**		***	
	$C_{c/c'}$	137.52	m	118.09	m	118.05	m
	$C_{d/d'}$	134.09	m	134.63	m	143.80<br>134.27	m<br>m
	$C_{e/e'}$	127.68	m	127.55	m	127.55	m
	$C_{f/f'}$	128.13	m	131.13	m	131.04	m
**Lys**	CH(α)	55.39	s	55.59	s	55.52	s
	CH2(β)	32.74	s	32.87	s	32.84	s
	CH2(γ)	14.77	s	15.56	s	15.56	s
	CH2(δ)	28.76	s	29.47	s	29.54	-
	CH2(ε)	38.46	s	38.98	s	38.91	s
**Arg**	CH(α)	51.87	s	51.32	s	51.37	s
	CH2(β)	27.29	s	24.66	s	24.32	s
	CH2(γ)	25.35	s	27.14	s	27.09	s
	CH2(δ)	40.86	s	41.04	s	41.04	s
	CN(ε)	*		**		***	
**Gly**	CH(α)	43.11	s	43.11	s	43.09	s
**Asp**	CH(α)	49.39	s	49.12	s	49.21	s
	CH2(β)	38.38	s	38.77	s	38.63	s
	CO(γ)	*		**		***	
**Phe**	CH(α)	53.65	s	53.36	s	53.37	s
	CH2(β)	36.91	s	36.86	s	36.81	s
	C(s)	138.77	s	not found		not found	
	CH(o/m)	128.06<br>129.34	s<br>s	129.31<br>128.02	s<br>s	129.31<br>128.02	s<br>s
	CH(p)	126.05	s	125.99	s	125.99	s

*For DP-RGD, 11 signals are observed in the 158.3-172.9 region, accounting for the eight carbonyl groups ($C_{a/a'}$, $C_{b/b'}$, 5 peptide bond carbonyls), two alkene carbons ($C_{b/b'}$) and Arg C_{ε}.

**For *trans*-[ReO$_2$(DP-RGD)$_2$]$^+$, signals for C=X, where X = O, N or C are weak, although >9 signals are observed in the 158.21-172.63 ppm region.

***For *cis*-[ReO$_2$(DP-RGD)$_2$]$^+$, signals for C=X, where X = O, N or C are weak, although multiple signals are observed in the 158.03-185.17 ppm region.

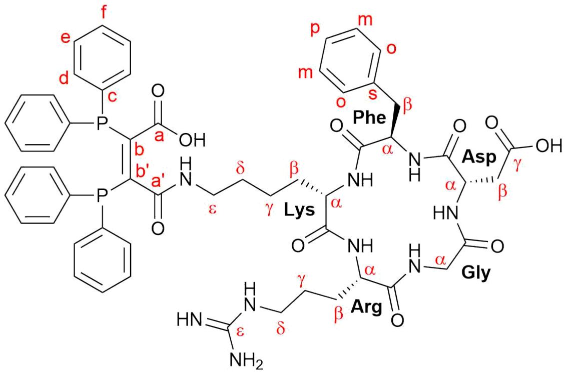

Table S3: Biodistribution of ^{99m}Tc in healthy balb/c mice (n = 4) administered either $[^{99m}TcO_2(DP\text{-}RGD)_2]^+$ or $[^{99m}TcO_2(DP\text{-}RGD)_2]^+$ + RGD peptide (to inhibit $\alpha_v\beta_3$ integrin uptake of radiotracer)

**Organ/tissue**	**1 h PI, %ID g^{-1} (± SD)**	**With block, 1 h PI, %ID g^{-1} (± SD)**	**Mean difference, %ID g^{-1}**	***p* value**
Blood	1.57±0.21	1.61±0.42	0.04	8.8×10^{-1}
Heart	1.92±0.10	0.90±0.17	1.02	**2.5×10^{-4}**
Lungs	7.55±1.44	5.50±0.86	2.05	5.1×10^{-2}
Liver	7.42±0.91	4.07±0.36	3.35	**4.8×10^{-4}**
Spleen	3.64±0.45	1.31±0.12	2.33	**5.9×10^{-5}**
Pancreas	1.02±0.11	0.40±0.05	0.62	**4.1×10^{-5}**
Stomach	4.77±0.58	1.20±0.24	3.57	**2.8×10^{-5}**
Small intestine	4.41±0.45	0.99±0.04	3.57	**5.0×10^{-6}**
Large intestine	2.24±0.20	0.53±0.11	3.42	**6.0×10^{-6}**
Kidneys	17.11±2.07	15.25±1.43	1.71	1.9×10^{-1}
Muscle	0.93±0.15	0.41±0.10	1.86	**1.1×10^{-3}**
Bone	1.73±0.55	0.89±0.17	0.52	2.7×10^{-2}
Skin+fur	2.48±0.88	2.02±0.30	0.46	3.5×10^{-1}
